# CXC receptor-4 mRNA silencing abrogates CXCL12-induced migration of colorectal cancer cells

**DOI:** 10.1186/1479-5876-9-22

**Published:** 2011-02-24

**Authors:** Claudia Rubie, Vilma O Frick, Pirus Ghadjar, Mathias Wagner, Christoph Justinger, Sabrina K Faust, Benjamin Vicinus, Stefan Gräber, Otto Kollmar, Martin K Schilling

**Affiliations:** 1 Department of General -, Visceral-, Vascular - and Paediatric Surgery, University of the Saarland, 66421 Homburg/Saar, Germany; 2 Department of Radiation Oncology, Inselspital, Bern University Hospital and University of Bern, 3010 Bern, Switzerland; 3 Institute of Pathology, University of the Saarland, 66421 Homburg/Saar, Germany; 4 Institute of Medical Biometrics, Epidemiology, and Medical Informatics (IMBEI) University of the Saarland, 66421 Homburg/Saar, Germany

## Abstract

**Background:**

Interactions between CXCR4 and its ligand CXCL12 have been shown to be involved in cancer progression in colorectal cancer (CRC). We performed a comparative CXCL12/CXCR4 expression analysis and assessed the effect of external CXCL12 stimulation on migration of CRC cells without and with CXCR4 inhibition.

**Methods:**

Expression of CXCL12/CXCR4 was assessed by quantitative real-time PCR, ELISA and immunohistochemistry in resection specimens of 50 CRC patients as well as in the corresponding normal tissues and in three human CRC cell lines with different metastatic potential (Caco-2, SW480 and HT-29). Migration assays were performed after stimulation with CXCL12 and CXCR4 was inhibited by siRNA and neutralizing antibodies.

**Results:**

In CRC tissues CXCL12 was significantly down-regulated and CXCR4 was significantly up-regulated compared to the corresponding normal tissues. In cell lines CXCR4 was predominantly expressed in SW480 and less pronounced in HT-29 cells. CXCL12 was only detectable in Caco-2 cells. CXCL12 stimulation had no impact on Caco-2 cells but significantly increased migration of CXCR4 bearing SW480 and HT-29 cells. This effect was significantly abrogated by neutralizing anti-CXCR4 antibody as well as by CXCR4 siRNAs (P < 0.05).

**Conclusions:**

CXCR4 expression was up-regulated in CRC and CXCL12 stimulation increased migration in CXCR4 bearing cell lines. Migration was inhibited by both neutralizing CXCR4 antibodies and CXCR4 siRNAs. Thus, the expression and functionality of CXCR4 might be associated with the metastatic potential of CRC cells and CXCL12/CXCR4 interactions might therefore constitute a promising target for specific treatment interventions.

## Background

Colorectal cancer (CRC) represents one of the most frequent malignancies worldwide with distant recurrence primarily affecting the liver as the predominant cause of CRC related mortality. The 5-year survival rate of 90% in patients with tumor restricted to the colon decreases to 10% in the presence of distant metastasis [1].

Recently, various cancer-related studies demonstrated that specific chemokines and their receptors may be involved in the molecular mechanisms that control metastasis in the early stages of cancer development [2]. In this respect, the homeostatic chemokine CXCL12, a non-ELR^+ ^CXC chemokine, has been implicated in promoting angiogenesis and metastasis [3,4]. CXCL12, also known as stromal derived factor 1 (SDF-1), is the only chemokine that is essential for survival [5] and a highly efficacious chemoattractant for T cells and thymocytes [6,7]. It is expressed by stromal cells such as fibroblasts and endothelial cells and signals exclusively via its G-protein-linked transmembrane receptor CXCR4 [8]. Expression of functional CXCR4 has been reported in various types of cancer cells [9-12], but also in immune cells such as peripheral blood lymphocytes, unprimed T cells, dendritic cells and lymphocytic leukemia B cells, where CXCR4 mediates spontaneous migration beneath bone marrow and stromal cells [13]. In infectious disease CXCR4 is employed by the human immunodefiency virus (HIV) to gain entry to cells [14] and in stem cell localization CXCR4 plays an important role for B-cell lymphopoiesis and bone marrow myelopoiesis [5]. In breast cancer, CXCR4 signaling was shown to play a crucial role in distant recurrence by mediating actin polymerisation and pseudopodia formation, thus, leading to chemotactic and invasive responses [3].

Recently, CXCR4 expression was associated with advanced tumor stage and the development and recurrence of lymphatic or distant colorectal liver metastases (CRLM) [15-17]. Thus, it was reported that CXCR4 is differentially expressed in CRC and significantly correlates with survival, recurrence and liver metastasis [15,18]. Moreover, it was shown that concomitant and high expression of CXCR4 and VEGF is a strong and independent predictor of early distant relapse in CRC [19] and recent evidence indicates that CXCR4 may also play a role in tumor angiogenesis of CRC [20].

Despite increasing knowledge about the expression of CXCL12/CXCR4 in CRC and its involvement of CXCR4 in the invasion and dissemination of CRC the precise mechanisms of CXCL12/CXCR4 interactions and subsequent metastasis are not entirely known. We therefore performed a comparative CXCL12/CXCR4 expression analysis and investigated how external addition of CXCL12 would promote CXCR4-mediated migration of CRC cell lines with different metastatic capabilities and how inhibition of CXCR4 by either CXCR4 siRNAs or neutralizing anti-CXCR4 antibodies would influence their migration potential.

## Methods

### Materials and patients

Surgical specimens and corresponding normal tissue from the same samples were collected from patients who underwent surgical resection at our department between 2003 and 2006. No patient underwent any specific cancer therapy prior to the resection. Our patient collectives comprised patients with CRC of different cancer stages (n = 50). In cases of organ confined CRC patients underwent resection of the primary tumor. Adjacent, disease free tissue samples of the colon and rectum served as control groups, respectively. Further, 10 specimens from patients with liver ruptures, focal nodular hyperplasia and haemangiomas as well as 10 unaffected tumor neighbor tissues from primary esophageal, pancreatic, gastric and colorectal carcinoma, respectively, were assessed. Informed consent for tissue procurement was obtained from all patients. The study was approved by the ethics commission of the Ärztekammer of the Saarland. The clinical variables presented in Table 1 were obtained from the clinical and pathological records and are in accordance with the UICC/TNM classification [21]. Cell culture assays were performed on non-metastatic cell line Caco-2 and metastatic cell lines SW480 and HT-29. Their metastatic potential has been investigated in murine liver metastasis models [22-24].

**Table 1:** Clinical characteristics of patients with colorectal carcinomas

Factor	**CRC**^**2 **^***n *= 50**
Localization of primary tumor	
Colon	23
Rectum	27
Gender	
Male	30
Female	20
Age, years^3^	63.9 (47-78)
Hepatitis (A,B or C)	
Positive	6
Negative	44
Largest tumor diameter (cm)^3^	4.7 (1.2-9.1)
TNM^1 ^stage of primary tumor	
I	8
II	16
III	16
IV	10
Grading	
I	0
II	22
III	27
Lymphatic permeation	
Positive	28
Negative	22
Vascular invasion	
Positive	7
Negative	43

^1^Tumor-node-metastasis; ^2^Colorectal carcinoma;

^3^Median with range in parentheses.

### Tissue preparation

Tissue samples were collected immediately after resection, snap frozen in liquid nitrogen and then stored at -80°C until they were processed under nucleic acid sterile conditions for RNA and protein extraction. Tumor samples were taken from vital areas of histopathologically confirmed adenocarcinomas. As corresponding normal tissue we used adjacent unaffected mucosa, 2-3 cm distal to the resection margin from the same resected adenocarcinoma. All tissues obtained were reviewed by an experienced pathologist (M.W.) and examined for the presence of tumor cells. As minimum criteria for usefulness for our studies we only chose tumor tissues in which tumor cells occupied a major component (>75%) of the tumor sample.

### Single-strand cDNA synthesis

Total RNA was isolated using RNeasy columns from Qiagen (Hilden, Germany) according to the manufacturer's instructions. RNA integrity was confirmed spectrophotometrically and by electrophoresis on 1% agarose gels. For cDNA synthesis 5 μg of each patient total RNA sample were reverse-transcribed in a final reaction volume of 50 μL containing 1× TaqMan RT buffer, 2.5 μM/L random hexamers, 500 μM/L each dNTP, 5.5 mM/L MgCl_2_, 0.4 U/μl RNase inhibitor, and 1.25 U/μL Multiscribe RT. All RT-PCR reagents were purchased from Applied Biosystems (Foster City, CA). The reaction conditions were 10 min at 25°C, 30 min at 48°C, and 5 min at 95°C.

### Real-time PCR

All Q-RT PCR assays containing the primer and probe mix were purchased from Applied Biosystems, (Applied Biosystems, Foster City, CA) and utilized according to the manufacturer's instructions. PCR reactions were carried out using 10 μL 2× Taqman PCR Universal Master Mix No AmpErase^® ^UNG and 1 μL gene assay (Applied Biosystems, Foster City, CA), 8 μL Rnase-free water and 1 μL cDNA template (50 mg/L). The theoretical basis of the qRT assays is described in detail elsewhere [25]. All reactions were run in triplicate along with no template controls and an additional reaction in which reverse transcriptase was omitted to assure absence of genomic DNA contamination in each RNA sample. For the signal detection, ABI Prism 7900 sequence detector was programmed to an initial step of 10 min at 95°C, followed by 40 thermal cycles of 15 s at 95°C and 10 min at 60°C and the log-linear phase of amplification was monitored to obtain C_T _values for each RNA sample.

Gene expression of all target genes was analyzed in relation to the levels of the slope matched housekeeping genes phosphomannomutase (PMM1) and β2-Microglobulin (β2 M) [26]. Data analysis was performed according to the relative standard curve method. Data are presented in relation to the respective housekeeping genes.

### Isolation of total protein

Protein lysates from frozen tissues were extracted with the radioimmunoprecipitation (RIPA) cell lysis and extraction buffer from Pierce (Pierce, Rockford, USA). Total protein content was assessed by using the Pierce BCA protein assay reagent kit (Pierce, Rockford, USA).

### Sandwich-Type Enzyme-Linked Immunosorbent Assay

The chemokine protein levels in the different tissue lysates were determined by sandwich-type enzyme-linked immunosorbent assays (ELISA) according to the manufacturer's instructions. Samples were assayed in duplicate with all values calculated as the mean of the two measurements. CXCL12 levels were assayed using a validated commercial ELISA (Duo Set R&D Systems, DY350, Minneapolis, Minn., USA). The absorbance was read at 450 nm in a 96-well microtiter plate reader. The chemokine concentration from each tissue lysate was normalized to the total protein content of each sample.

### Immunohistochemistry/Immunocytochemistry

Operative specimens were routinely fixed in formalin and subsequently embedded in paraffin. Before staining, 4-μm thick paraffin-embedded tissue sections were mounted on Superfrost Plus slides, deparaffinized and rehydrated in graded ethanol to deionized water. The sections were microwaved with an antigen retrieval solution (Target Retrieval, Dakocytomation, Carpinteria, CA, USA) and after blocking of endogenous peroxidase activity with 3% hydrogen peroxide, the sections were sequential treated with avidin and biotin (Avidin/Biotin blocking kit, Vector Laboratories Inc., Burlingame, CA, USA). In addition the specimens were further blocked for 30 min at room temperature with normal rabbit serum. Overnight incubation at 4°C with primary goat polyclonal anti-human CXCR4 antibody (1:300, AB1671, Abcam, Cambridge, UK) was followed by incubation of secondary biotinylated rabbit anti-goat IgG antibody and the avidin-biotin-peroxidase reaction (Vectastain ABC ELITE Kit, Vector Laboratories, Burlingame, CA, USA). After colour reaction with aminoethylcarbazol solution (Merck, Darmstadt, Germany), tissues were counterstained with haematoxylin. Immunocytochemical staining of CRC cells was performed using culture slides from Becton Dickinson (Becton Dickinson, Heidelberg, Germany). After blocking of endogenous peroxidase activity and blocking for 30 min at room temperature with normal rabbit serum, cells were overnight incubated at 4°C with primary goat polyclonal anti-human CXCR4 antibody (1:300, AB1671, Abcam, Cambridge, UK). Following incubation of secondary biotinylated rabbit anti-goat IgG antibody and the avidin-biotin-peroxidase reaction (Vectastain ABC ELITE Kit, Vector Laboratories, Burlingame, CA, USA) and all further steps were carried out as described above for the immohistochemical staining procedure.

Negative controls were conducted in all cases omitting primary antibody. For evaluation of immunocytochemical staining the total number of cells per 5 high-power fields (using ×40 -HPF objective magnification) was determined. Cells were considered positive, when they demonstrated strong and exclusive labelling.

### Cell migration assays

Cell migration assays were performed in triplicate using BD Falcon cell culture inserts containing polyethylene terephthalate membranes (8 μm pore size) from BD Biosciences (Bedford, MA, USA). Caco-2, HT-29 and SW480 cells at a concentration of 50000 cells per ml in 500 μl medium with antibiotics but without FCS were placed in the top of a two-chamber assay system and incubated for 48 hours with and without CXCL12 (350-NS-10, R&D Systems, Wiesbaden, Germany) in 800 μl medium without FCS placed in the lower chamber. CXCL12 concentrations in the receiver wells were 10 ng, 50 ng and 100 ng per ml, respectively. After the incubation period, the non-migrated cells on the upper surface of the filters were removed using a cotton swab. Migrated cells, which are adherent to the lower surface of the filters, were stained with a 0.1% crystal violet solution (Merck, Darmstadt, Germany). The number of these migrated cells was quantified microscopically by counting them in 10 high-power microscopic fields. Medium with 20% FCS placed in the lower chamber of the two-chamber assay system served as a positive control and reference in the migration assays.

### Cell proliferation assays

Cell proliferation assays (Roche Diagnostics GmbH, Mannheim, Germany) were performed in triplicate using flat-bottomed 96-well microtiter plates for culturing Caco-2, HT-29 and SW480 cells in the presence of CXCL12 at 37°C for 48 hours. Subsequently, bromodeoxyuridine (BrdU) was added to the cells and the cells were reincubated for 24 hours. During this labeling period the pyrimidine analogue BrdU is incorporated in place of thymidine into the DNA of proliferating cells. After removing the culture media, cells were fixed and anti-BrdU-POD was added to bind to the BrdU incorporated in newly synthesized cellular DNA. Immune complexes were detected by the subsequent substrate reaction and the reaction product was quantified by photometrically measuring the absorbance of the developed color.

### Cell inhibition assays

Inhibition assays were performed in analogy to the cell migration assays using BD Falcon cell culture inserts from BD Biosciences. Similar to the migration assays Caco-2, HT-29 and SW480 cells in 500 μl medium without FCS were mixed with anti-CXCR4 antibodies (MAB171, R&D systems) applied in a final concentration of 6 μg per ml and placed in the upper chamber of a two-chamber assay system. After incubation for 48 hours with and without CXCL12 in 800 μl medium without FCS placed in the lower chamber steps were continued as mentioned above for the migration assays and migrated CRC cells were quantified microscopically by counting the cells that had migrated into the filters.

### siRNA assays

CXCR4 siRNA transfection of Caco-2, HT-29 and SW480 cells was performed according to the HiPerFect Transfection Reagent Handbook from Qiagen (Qiagen, Hilden, Germany) and the Dharmacon DharmaFECT siRNA transfection protocol (Thermo Fisher Scientific, Waltham, USA). Briefly, cells were trypsinized, counted and then diluted in antibiotic-free complete medium containing serum to achieve the appropriate plating density in 100 μl of solution. On average, 6 × 10^4 ^cells per well of a 24-well plate or 5 × 10^5 ^cells per well of a 6-well plate, respectively, were seeded and overnight incubated under their normal growth conditions. On the day of transfection, four different CXCR4 siRNAs (Qiagen, SI00052220 (1), SI00052227 (2), SI02664235 (7), SI02664242 (8)) were diluted to a final concentration of 10 nM in 100 μl culture medium without serum, respectively. 3 μl of HiPerFect (Qiagen) for the transfection of SW480 cells and 5 μl of Dharmacon DharmaFECT siRNA transfection reagent (Thermo Fisher Scientific) for the transfection of Caco-2 and HT-29 cells were added to the diluted siRNA reactions, respectively. Samples were incubated for 5-10 minutes at room temperature to allow formation of transfection complexes. Hence, the complexes were added drop-wise to the cells and incubated for three hours under their normal growth conditions. Gene silencing was monitored after 48 and 72 hours after transfection at the mRNA and at the protein level using Realtime PCR and western blotting, respectively. Further, gene silencing was monitored visually by observing the effect on a cell death control. Further, control samples with untransfected cells, a mock-transfection with only transfection reagent, a negative control siRNA and control samples with MAPK1 siRNA were used. A detailed overview of siRNA control experiments is presented in Table 2. Subsequent migration assays were performed as described above.

**Table 2:** Applied RNAi control experiments - overview

Control experiment	Type	Example
Positive control siRNA	siRNA that is known to provide high knockdown of its target gene	Hs_MAPK1 siRNA (Qiagen) OrderNo. 1022564
Negative control siRNA	a nonsilencing siRNA with no homology to any known mammalian gene	AllStars Negative control siRNA (Qiagen) OrderNo. 1027280
Transfection control siRNA	siRNA that is used to measure the transfection efficiency, e.g. by siRNA	AllStars Hs Cell Death Control siRNA (Qiagen) OrderNo. 1027298
Mock transfection control	control experiment where cells go through the transfection process without addition of siRNA	
Untransfected cells control	control experiment where gene expression analysis is carried out on cells that have not gone through the transfection process	

### Western Blot Analysis

Gene silencing was monitored 72 hours after transfection at the protein level using western blotting. Total protein (25 μg/lane) was separated by SDS-PAGE using a 10% gel and blotted onto nitrocellulose membranes (Hybond ECL, Amersham Biosciences, Piscataway, NJ, USA). Membranes were blocked by incubation in Tris-buffered saline (TBS) containing 5% non fat dry milk and 0.1% Tween 20 for 2 h at room temperature and then incubated overnight at 4°C with primary goat polyclonal anti-human CXCR4 antibody (1:300, AB1671, Abcam, Cambridge, UK). Blots were then washed and incubated at room temperature for 1 h with donkey anti-goat HRP antibody (diluted 1:5000, sc-2056, Santa Cruz Biotechnology, Santa Cruz, CA USA). Bands were visualized by ECL Western blotting analysis systems (Amersham Biosciences, Piscataway, NJ, USA). The human cell lysate HL-60 (sc-2209, Santa Cruz Biotechnology, Santa Cruz, CA, USA) served as positive control. Quantification of band intensities has been performed on three independent samples using image J software.

### Calculations and Statistical Methods

Chemokine receptor expression profiles in the different groups are shown as mean and standard error of the mean (SEM). Statistical calculations were done with the MedCalc software package. Where appropriate, either the Student's t-test or the Wilcoxon's rank sum test was applied to test for group differences of continuous variables. A *P *value of 0.05 or less was considered significant.

## Results

### CXCL12/CXCR4 expression in CRC tissues

**Figure 1:**
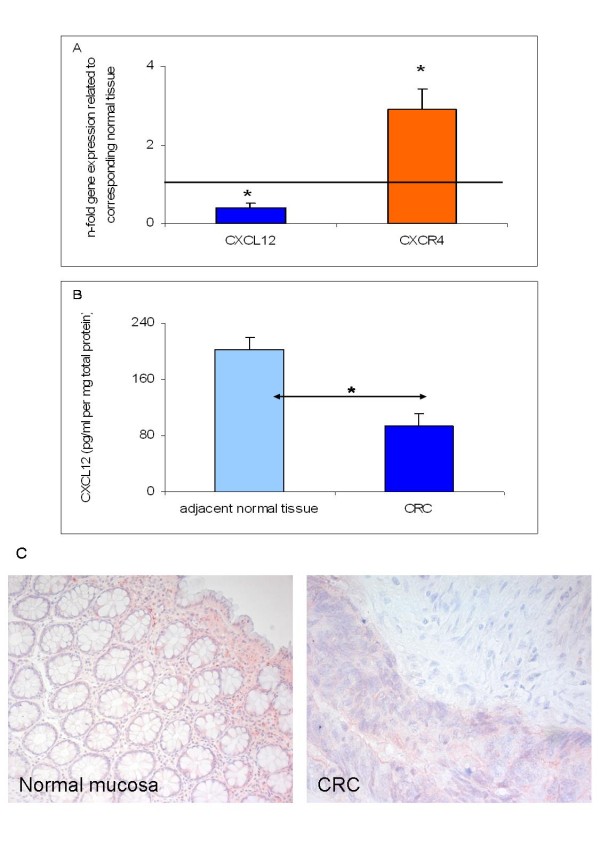
**CXC12/CXCR4 expression in colorectal cancer (CRC) tissue specimens as determined by (A) Q-RT-PCR for CXCL12 and CXCR4, (B) ELISA analysis for CXCL12 and (C) immunohistochemistry for CXCR4**. (A) Q-RT-PCR data are expressed as mean +/- standard error of the mean (SEM), *P < 0.05, *n *= 50. Fold increase above 1 indicates gene overexpression in affected tissues related to unaffected neighbor tissues. (B) Detection of CXCL12 protein concentrations (pg/ml pro mg total protein) in total cell lysates of CRC and adjacent normal tissues from CRC patients (*n *= 50). Protein data are expressed as mean +/- SEM, *P < 0.05. (C) Detection of CXCR4 protein expression in representative CRC specimens as assessed by immunohistochemical staining with CXCR4-specific antibodies showing positive cytoplasmic staining in CRC and in unaffected corresponding tissues (original magnification × 200 and × 400).

Q-RT-PCR analysis of CRC tissue specimens revealed significant up-regulation of CXCR4 and significant down-regulation of CXCL12 compared to corresponding normal tissues (Figure 1A). These results were verified for CXCL12 on the protein level as shown by ELISA assays which demonstrated significant CXCL12 down-regulation in CRC tissues compared to the corresponding normal tissues (Figure 1B). Detection of CXCR4 expression was assessed by immunohistochemical staining. Immunostaining of the CRC tissues revealed positive cytoplasmic staining in 28 out of 50 CRC specimens (56%) as shown for a representative example in Figure 1C. However, no significant difference in CXCR4 expression was detected according to the metastatic behaviour. Patients with synchronous or metachronous colorectal liver metastases expressed CXCR4 in their primary tumor with no significant difference with respect to CRC patients who did not develop metastases.

### Organ-specific expression of CXCL12

**Figure 2:**
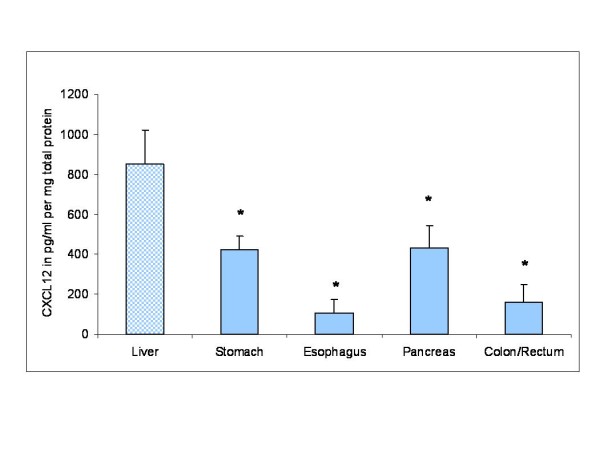
**CXCL12 protein expression in different human organs related to the liver as determined by ELISA**. CXCL12 protein concentrations (pg/ml per mg total protein) were measured in total cell lysates of normal liver, pancreas, stomach, colon/rectum and esophagus tissues of 10 patients, respectively (*n *= 10). Protein data are expressed as mean +/- SEM, *P < 0.05.

CXCL12 mRNA and protein expression as determined by RT-PCR and ELISA was investigated in different human organs such as pancreas, stomach, colon/rectum and esophagus related to the liver. On the protein level CXCL12 was found to exhibit peak levels of expression in the liver compared to stomach, esophagus, pancreas, colon and rectum (Figure 2). However, on the mRNA level CXCL12 did not show a markedly higher expression in the liver in comparison to other gastrointestinal organs or glands corresponding to previous results [27].

### CXCL12/CXCR4 expression in CRC cell lines

**Figure 3:**
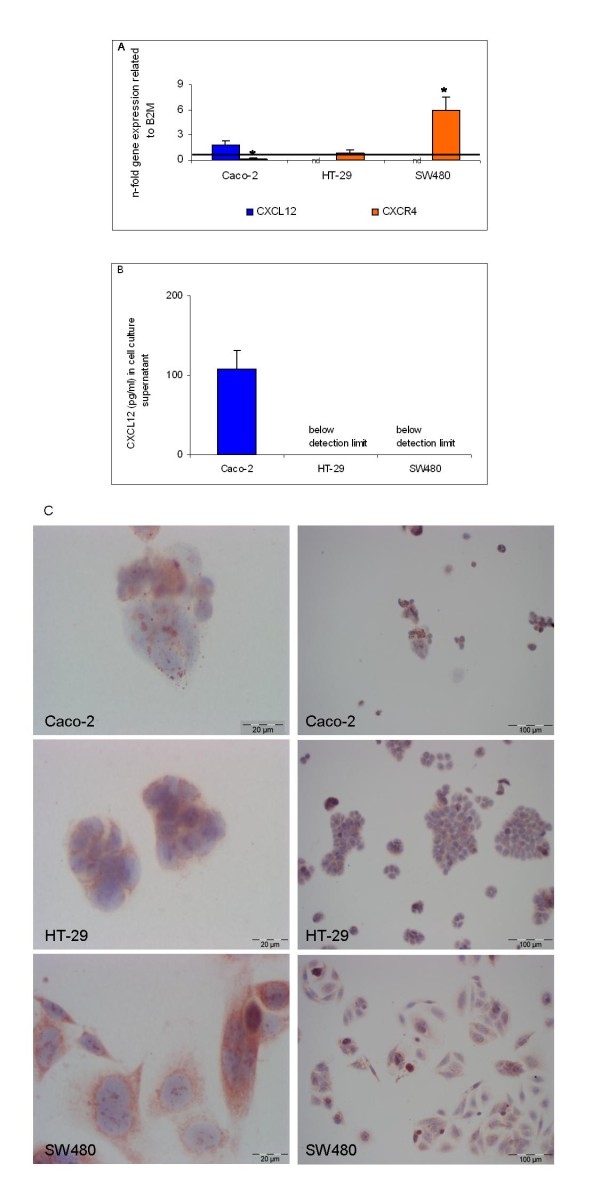
**CXC12/CXCR4 expression in Caco-2, HT-29 and SW480 cells as determined by (A) Q-RT-PCR for CXCL12 and CXCR4, (B) ELISA analysis for CXCL12 and (C) immunocytochemistry for CXCR4**. (A) Q-RT-PCR data are expressed as mean +/- standard error of the mean (SEM), *P < 0.05. Fold increase above 1 indicates gene overexpression related to housekeeping gene B2M. (B) Detection of CXCL12 protein concentrations in pg/ml in cell culture supernatant of Caco-2, HT-29 and SW480 cells. Protein data are expressed as mean +/- SEM. (C) Detection of CXCR4 protein expression in representative cell culture slides of Caco-2, HT-29 and SW480 cells as assessed by immunocytochemical staining with CXCR4-specific antibodies.

In non-metastatic cell line Caco-2 low CXCL12 expression was detected on the mRNA level by PCR and on the protein level in the cell culture supernatant by ELISA (Figure 3A and 3B, respectively). In metastatic cell lines SW480 and HT-29 CXCL12 mRNA and protein expression was below detection limit (Figure 3A and 3B, respectively). In contrast, CXCR4 mRNA expression was detected in HT-29 and SW480 cells with the latter displaying significant overexpression relative to B2M (Figure 3A). When CXCR4 protein expression was assessed by immunocytochemical staining, CXCR4 positive staining was observed in 22% of Caco-2 cells, 74% of HT-29 cells and 80% of SW480 cells as quantified microscopically and shown for a representative example in Figure 3C. Likewise, CXCR4 protein expression as determined by western blot analysis revealed similar expression rates in HT-29 and SW480 cells. Variation in CXCR4 mRNA and protein data may be due to posttranscriptional and posttranslational modifications as well as to the relative amplification modus of the qRT-PCR results with respect to housekeeping gene B2M.

### CXCR4 protein inhibition abrogates migration of colorectal cancer cells

**Figure 4:**
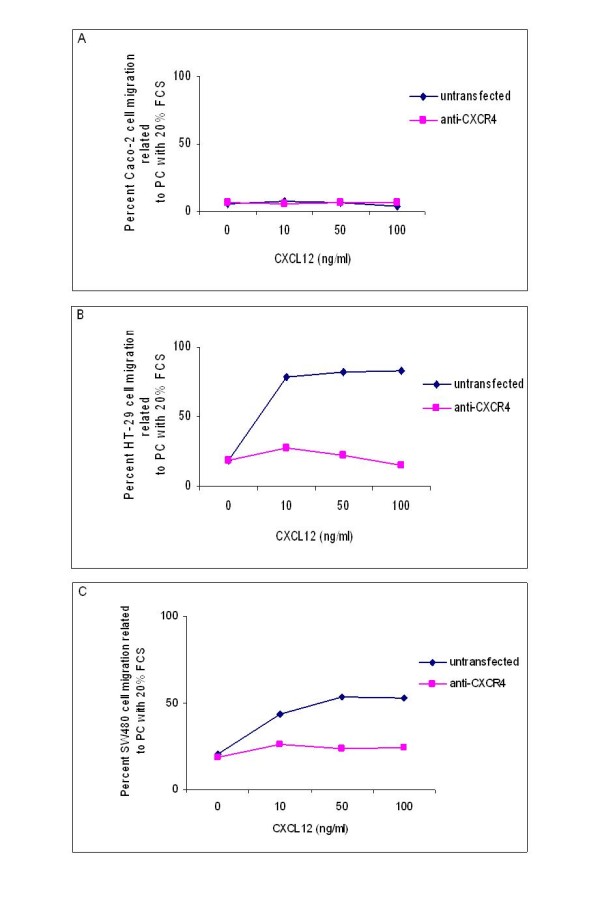
**CXCR4 protein inhibition abrogates migration of colorectal cancer cells**. (A) Percent Caco-2 cell migration related to a positive control containing 20% FCS (PC) without inhibition and after inhibition with anti-CXCR4 antibody. (B) Percent HT-29 cell migration related to PC without inhibition and after inhibition with anti-CXCR4 antibody. (C) Percent SW480 cell migration related to PC without inhibition and after inhibition with anti-CXCR4 antibody.

All three cell lines were stimulated with 10, 50 and 100 ng/ml CXCL12, respectively, and incubated for 48 hours. While Caco-2 cells were not stimulated by any concentration of CXCL12 (Figure 4A), we observed a significant CXCL12 dose-independent stimulation of migration for HT-29 and SW480 cells (Figure 4B and 4C, respectively) (*P *< 0.05). Thus, CXCL12 was shown to be chemotactic for HT-29 and SW480 cells.

When we added neutralizing anti-CXCR4 antibody prior to performance of cell migration assays, the CXCL12 stimulated migration of HT-29 and SW480 cells was significantly abrogated at all CXCL12 concentrations under investigation (Figure 4B and 4C, respectively) (*P *< 0.05). Application of anti-CXCR4 antibodies had no impact on the migration potential of Caco-2 cells (Figure 4A).

### CXC receptor-4 gene silencing abrogates migration of colorectal cancer cells

**Figure 5:**
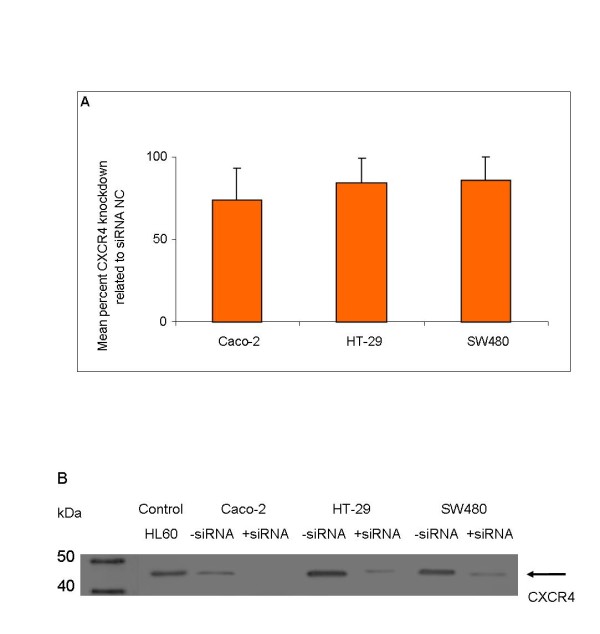
**Demonstration of CXCR4 knockdown as determined by mRNA and protein analysis**. (A) Mean percent CXCR4 knockdown related to siRNA negative control (NC) as determined by mRNA analysis in Caco-2, HT-29 and SW480 cells. (B) CXCR4 protein expression after CXCR4 siRNA silencing as determined by Western Blot analysis in Caco-2, HT-29 and SW480 cells related to untransfected total cell lysates. Total cell lysates of untransfected cells and one representative example of CXCR4 siRNA transfected cells of each cell line were immunoblotted with antibodies specifically recognizing chemokine receptor CXCR4. Acute leucemia cell line HL60 served as positive control for the detection of CXCR4.

**Figure 6:**
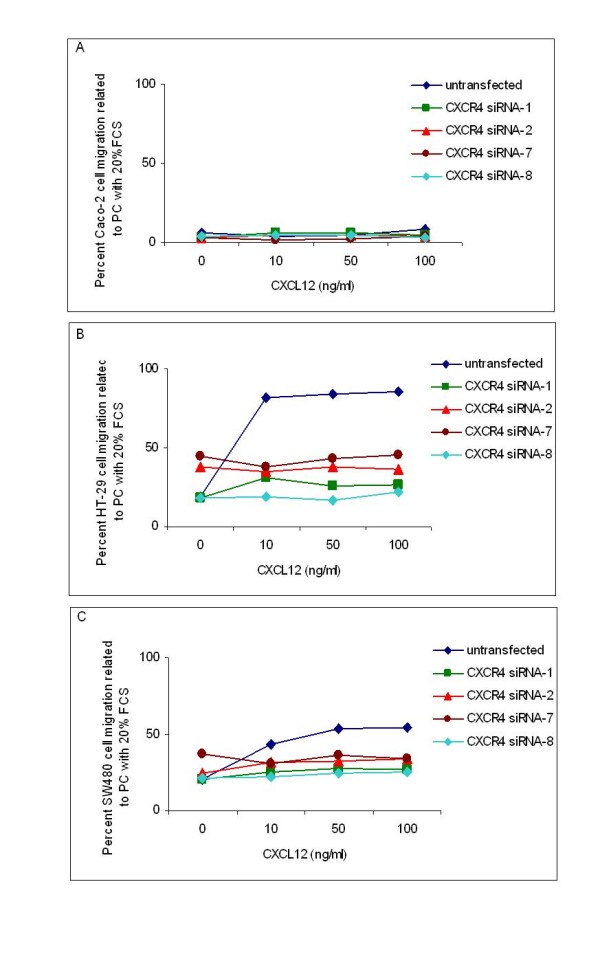
**CXCR4 mRNA silencing abrogates migration of colorectal cancer cells**. (A) Percent Caco-2 cell migration related to positive control containing 20% FCS (PC) without transfection and after transfection with 4 different CXCR4 siRNAs. (B) Percent HT-29 cell migration related to PC without transfection and after transfection with 4 different CXCR4 siRNAs. (C) Percent SW480 cell migration related to PC without transfection and after transfection with 4 different CXCR4 siRNAs.

To analyze if the chemotactic effect of CXCL12 on the migration of HT-29 and SW480 cells could be counteracted by the down-regulation of the mRNA of corresponding receptor CXCR4, we applied four different CXCR4 siRNAs. The mean percent CXCR4 knockdown related to the siRNA negative control was 78% for Caco-2 cells, 80% HT-29 cells and 84% for SW480 cells as determined by RT-PCR 48 hours after transfection on the mRNA level (Figure 5A). On the protein level gene silencing was monitored 72 hours after transfection in western blot analyses (Figure 5B). Application of CXCR4 siRNAs had no impact on the migration potential of Caco-2 cells (Figure 6A). In contrast, the CXCL12 stimulated migration of HT-29 and SW480 cells was significantly abrogated by all four different CXCR4 siRNAs at all CXCL12 concentrations under investigation (Figure 6B and 6C, respectively) (*P *< 0.05).

### Proliferative rate of colorectal cancer cells after CXCR4 blockage by mRNA silencing and inhibition antibodies

**Figure 7:**
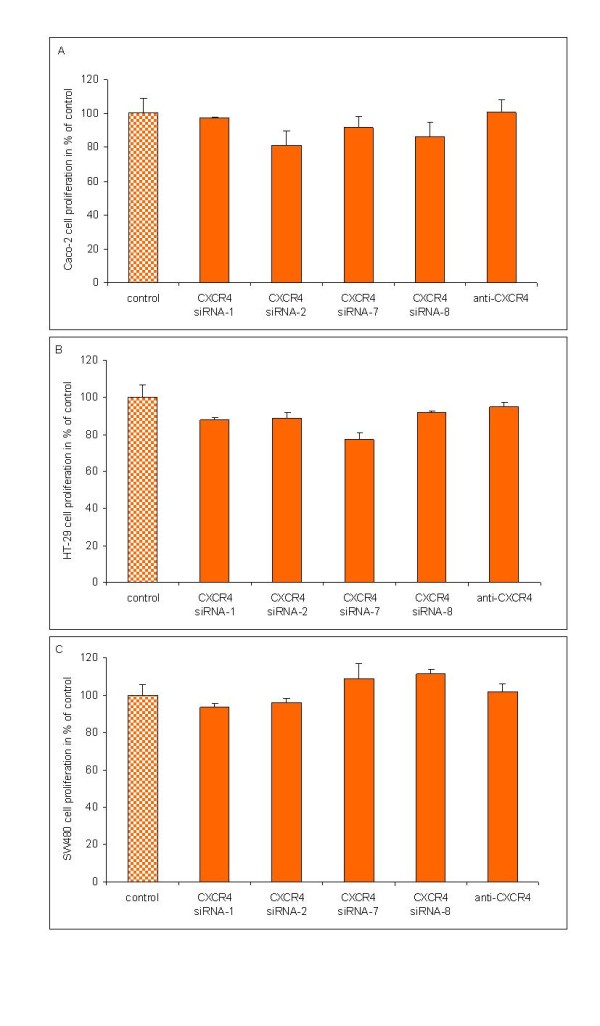
**Proliferative rate of colorectal cancer cells after CXCR4 mRNA silencing or CXCR4 blockage by inhibition antibodies. **Knockdown of endogeneous CXCR4 expression by four different CXCR4 siRNAs or CXCR4 blockage by ant-CXCR4 inhibition antibodies does not decelerate the proliferation rate of (A) Caco-2 cells, (B) HT-29 cells and (C) SW480 cells in relation to untransfected cells without inhibition antibody (control).

CXCR4 blockage by mRNA silencing or anti-CXCR4 antibodies might restrain the proliferation of CRC cell lines, so that the decreased migration capacity of cancer cells might result from a lower proliferative rate. To ensure that the significantly abrogated migration capacity of HT-29 and SW480 cells after CXCR4 mRNA silencing by all four different CXCR4 siRNAs is not resulting from a lower proliferative rate with respect to the untransfected cells, we investigated their proliferative capacity before and after siRNA transfection. In addition, we also included cell line Caco-2 in this survey. Likewise, we investigated the proliferative capacity of Caco-2, HT-29 and SW480 cells before and after CXCR4 blockage by neutralizing anti-CXCR4 antibody. Here, cells without anti-CXCR4 antibody served as reference. As presented in figure 7 there is no marked difference between the proliferation rates of Caco-2 (Figure 7A), HT-29 (Figure 7B) and SW480 (Figure 7C) cells before or after CXCR4 mRNA silencing or CXCR4 blockage by anti-CXCR4 antibody. Thus, the significantly abrogated migration capacity of HT-29 and SW480 cells after CXCR4 mRNA silencing or CXCR4 blockage by anti-CXCR4 antibody is not resulting from a reduced proliferation rate.

## Discussion

The importance of the CXCL12/CXCR4 axis in tumor progression and metastasis has been emphasized. Recent evidence also suggests that the CXCL12/CXCR4 system is involved in the progression and metastasis of CRC [15,20,28].

Thus, the aim of this study was to conduct a comparative CXCL12/CXCR4 expression analysis and to investigate the functional role of CXCR4 in metastatic and non-metastatic CRC derived cell lines. For this purpose the migration potential of these cell lines was tested under conditions where CXCR4 was inhibited on the mRNA as well as on the protein level. On the mRNA level, CXCR4 silencing was achieved by CXCR4 RNA interference while on the protein level inhibition of CXCR4 protein activity was achieved using an anti-CXCR4 antibody.

As shown initially, CXCL12 protein is significantly higher expressed in the liver in relation to other intestinal organs or glands, thus supporting potential CXCL12 involvement in the homing of CRC cells to the liver. Subsequently, we observed significant up-regulation of CXCR4 and significant down-regulation of CXCL12 in CRC tissues. This inverse expression pattern is supported by previous studies, where CXCR4 was shown to be highly up-regulated in all T-stages of CRC tissues [16] and CXCL12 expression levels were shown to be low at the base of the crypts and increased in the more differentiated apical intestinal epithelial cells [29,30]. In compliance with the low CXCL12 expression status in tissues we observed low or no CXCL12 expression, respectively, in three human CRC derived cell lines, non-metastatic cell line Caco-2 and metastatic cell lines SW480 and HT-29. While low CXCL12 expression was detected in Caco-2 cells on the mRNA and on the protein level, CXCL12 was below the detection limit in HT-29 and SW480. It was hypothesized that changes in epithelial CXCL12 expression might contribute to CRC disease progression by allowing CRC cells to more readily sense CXCL12 from exogeneous sources hereby promoting metastasis [31]. Wendt et al. have shown that the reduced CXCL12 expression pattern in CRC tissues and cells is due to DNA hypermethylation in primary CRC and carcinoma-derived cell lines. Thus, it was demonstrated that inhibition or ablation of DNA methyltransferases prevent promoter methylation and restore CXCL12 expression. In addition, re-expression of functional, endogeneous CXCL12 in CRC cells was shown to reduce metastatic tumor formation significantly while silencing CXCL12 greatly enhanced the metastatic potential of CRC cells [31]. Moreover, endogeneous CXCL12 was shown to provide a barrier to metastasis by increasing anoikis via activation of a Bim-mediated intrinsic apoptotic pathway [32,33]. These results may constitute a plausible explanation for our observation of significantly reduced CXCL12 expression rates in CRC tissues and carcinoma-derived cell lines. Concordantly, an elevated migratory signaling response to ectopic CXCL12 was also shown to contribute to the metastatic potential of CXCR4-expressing mammary carcinoma cells, subsequent to epigenetic silencing of autocrine CXCL12 [34].

For CXCR4 we observed an inverse expression pattern in the three CRC-derived cell lines. Thus, expression in non-metastatic cell line Caco-2 was significantly lower compared to the metastatic cell lines under investigation. Also drug-resistant invasive HT-29 cells with a metastatic behaviour in immunodeficient mice were shown to exhibit high CXCR4 expression [35]. Responsible for promoting invasion in drug-resistant colon carcinoma cells is the autocrine CXCR4 ligand macrophage migration-inhibitory factor (MIF) as shown by Dessein et al. Impairing the MIF-CXCR4 signaling pathway, e.g. by silencing CXCR4, abolished this aggressive phenotype. To date, in various cancer entities aberrantly expressed chemokine receptors were demonstrated to contribute directly to the development of organ selective distant metastasis by interaction with their organ selectively expressed chemokine ligands. As a consequence of the low CXCR4 expression, Caco-2 cells showed no increase in migration in response to CXCL12 stimulation. In contrast, CXCL12 significantly increased cell migration in the CXCR4 expressing metastatic cell lines SW480 and HT-29. While a stimulative effect of CXCL12 on migration of CRC cell lines was described before [36], these effects were not significant and refer only to one CXCL12 concentration (100 ng/ml). Moreover, these tests were performed on CRC cell lines LS174T, SW620 and SW480 but not on cell lines HT-29 and Caco-2. To further investigate the functional role of CXCR4 we performed inhibition assays with anti-CXCR4 antibodies added previously to CXCL12 stimulation. The addition of the inhibition antibodies significantly blocked the CXCL12-dependent stimulation of HT-29 and SW480 cell migration but had no impact on Caco-2 migration. Our results correlate with inhibition studies performed on SW480 cells [37], where chemotaxis induced by CXCL12 was also shown to be blocked, although not as complete as we have shown for both SW480 and HT-29 cells. Likewise, Kim et al [38] reported on a partial inhibitory effect of anti-CXCR4 antibodies on CXCL12 stimulated melanoma and CRC cell migration (38%). However, to date no study analyzed the effect of CXCR4 gene silencing on CXCL12 mediated cell migration of CRC cells. Thus, we investigated if the chemotactic effect of CXCL12 on the migration of HT-29 and SW480 cells could be counteracted by the down-regulation of the CXCR4 mRNA. Applying four different CXCR4 siRNAs we achieved a mean percent CXCR4 knockdown of 78-84% and a significant abrogation of CXCL12 stimulated migration of HT-29 and SW480 for all four different CXCR4 siRNAs at all CXCL12 concentrations under investigation. On the other hand, application of CXCR4 siRNAs had no impact on the migration potential of Caco-2 cells. Thus, we have demonstrated the functional status of the CXCR4 receptor on CRC cell lines in response to CXCL12 on the mRNA level.

In CRC patients, no significant difference in CXCR4 expression was detected according to the metastatic behaviour. Based on our in vitro results CRC patients with metastasis may be expected to express more CXCR4 in their tumor cells compared to CRC patients without metastasis. However, many other important factors contribute to the metastatic properties of tumor tissues and influence their metastatic potential. When a CRC cell is turned metastatic, CXCR4 may be an important factor for the homing of such a tumor cell to its favourite organ destination, the liver.

Our results are well in line with recent findings demonstrating a role of CXCL12 in promoting cell migration and tumor growth of CRC metastasis *in vivo *in a murine model [39]. However, while we observed no CXCR4-dependent proliferation of CRC cells, Shen et al. demonstrated CXCR4-induced proliferation for pancreatic cancer cells, where it was linked to AKT and ERK dependent pathways [40]. Moreover, CXCR4 knockdown by small interfering RNA was shown to inhibit cell proliferation and invasion of oral squamous cell carcinoma cells [41]. A recent study demonstrated that CXCL12/CXCR4 interactions may also promote early extravasation of liver metastatic epithelial tumor cells which determines a critical step in formation of organ-specific metastases [42]. Currently, various small-molecule chemokine receptor antagonist compounds are undergoing development in phase I to III studies in infectious and autoimmune diseases and a CCR5 inhibitor is already in the clinic for the treatment of HIV-infected patients.

## Conclusions

In conclusion, our results provide evidence that CXCR4 is up-regulated in CRC and stimulation of CXCR4 bearing cancer cells with CXCL12 led to increased migration, an effect which could be inhibited both by CXCR4 siRNA and neutralizing CXCR4 antibodies. Interestingly, CXCR4 was predominantly expressed in cell lines with metastatic potential and consequently, these cell lines showed increased migration after external CXCL12 stimulation. Our results suggest that the metastatic potential of CRC cells may be associated with the aberrant expression of CXCR4 and subsequently the ability of cells to interact with CXCL12 via autocrine and/or paracrine mechanisms.

## Competing interests

The authors declare that they have no competing interests.

## Authors' contributions

All authors read and approved the final manuscript. CR is responsible for the study concept and design and drafted the manuscript. VOF took part in the acquisition, analysis and interpretation of the data. PG is responsible for the critical assessment and revision of the manuscript. MW examined the tissue sections for the presence of tumor cells and histopathologically confirmed all tissues under investigation. CJ provided clinical information and SG participated in the statistical analysis. SKF performed the siRNA experiments and BV contributed to scientific discussion. OK provided clinical information. MKS is responsible for the provision of all the patient material and participated in the critical revision of the manuscript for important intellectual content.
